# *Plutella australiana* (Lepidoptera, Plutellidae), an overlooked diamondback moth revealed by DNA barcodes

**DOI:** 10.3897/zookeys.327.5831

**Published:** 2013-08-29

**Authors:** Jean-François Landry, Paul DN Hebert

**Affiliations:** 1 Canadian National Collection of Insects, Arachnids, and Nematodes, Agriculture and Agri-Food Canada, Eastern Cereal and Oilseed Research Centre, C.E.F., Ottawa, ON K1A 0C6, Canada; 2 Biodiversity Institute of Ontario, University of Guelph, Guelph, ON N1G 2W1, Canada

**Keywords:** Australia, *karsholtella*, new species, *xylostella*

## Abstract

The genus *Plutella* was thought to be represented in Australia by a single introduced species, *Plutella xylostella* (Linnaeus), the diamondback moth. Its status as a major pest of cruciferous crops, and the difficulty in developing control strategies has motivated broad-ranging studies on its biology. Prior genetic work has generally supported the conclusion that populations of this migratory species are connected by substantial gene flow. However, the present study reveals the presence of two genetically divergent lineages of this taxonin Australia. One shows close genetic and morphological similarity with the nearly cosmopolitan *Plutella xylostella*. The second lineage possesses a similar external morphology, but marked sequence divergence in the barcode region of the cytochrome *c* oxidase I gene, coupled with clear differences in genitalia. As a consequence, members of this lineage are described as a new species, *Plutella australiana* Landry & Hebert, which is broadly distributed in the eastern half of Australia.

## Introduction

The diamondback moth, *Plutella xylostella* (Linnaeus), is one of the most damaging insect pests, attacking cruciferous crops, such as cabbage and cauliflower, across its nearly cosmopolitan range. Because biological agents have proven ineffectual (Goodwin 1979), most control programs for this moth have relied on insecticides. Costs for its control are significant; they were estimated at one billion US dollars in 1992 (Talekar and Shelton 1993). Because *Plutella xylostella* has rapidly developed resistance to most insecticides (Sun et al. 1986), and was the first insect species to become resistant to *Bacillus thuringensis* (Talekar and Shelton 1993), work is being directed towards gaining a deeper understanding of its biology. One line of investigation has involved evaluations of gene flow among its populations through the analysis of protein polymorphisms and sequence divergence in mitochondrial genes. Initial studies of allozyme variation (Caprio and Tabashnik 1992, Kim et al. 1999) provided little evidence for geographic shifts in gene frequencies. Subsequent analysis of sequence variation in the mitochondrial cytochrome *c* oxidase I (COI) gene in *Plutella xylostella* from China, Hawaii, Korea, Philippines, and USA showed less than 1% sequence divergence among populations (Chang et al. 1997, Li et al. 2006). These genetic results suggest substantial gene flow among widely separated populations, supporting expectations from observational studies which have indicated that *Plutella xylostella* is highly migratory with populations in cool temperate regions annually reestablished from southerly locales (Harcourt 1986, Chapman et al. 2002). However, another allozyme study on *Plutella xylostella* from five continents (Africa, Asia, Australia, Europe, North America) provided a slightly different perspective (Pichon et al. 2006). Populations from most sites had similar allele frequencies, but the population from Japan showed considerable differentiation from those at the other sites, while the Australian populations showed variability. Specimens collected near Sydney possessed allele frequencies similar to populations in other nations (except Japan), but those from four other sites were distinctive. Based on these results, Pichon et al. (2006) concluded that gene flow was sometimes insufficient to prevent regional genetic divergence.

The present study was motivated by a large-scale DNA barcode study of Australian Lepidoptera (Hebert et al. 2013) which indicated that specimens assigned to *Plutella xylostella* included two lineages with substantial sequence divergence in the barcode region of COI. The present analysis places these results in perspective by comparing levels of sequence divergence among other members of this genus, and by examining the morphology of the two Australian lineages. Because these results provide compelling evidence that the lineages represent different species, a new taxon, apparently endemic to Australia, is described.

## Materials and methods

### Collections

Most of the Australian specimens of *Plutella* examined in this study were collected at UV light from 2004–2012 as a result of a sampling program to obtain specimens of Lepidoptera for DNA barcode analysis. The results from Australian specimens were placed in a broader perspective through the inclusion of sequence records from two specimens (when available) for each of five other non-Australian *Plutella* species and one species in the closely allied genus *Eidophasia* possessing coverage on BOLD (Ratnasingham and Hebert 2007). In addition, a barcode record was obtained from the holotype of *Plutella karsholtella* Baraniak, a species which shows close morphological similarity to *Plutella xylostella*. Described from the Canary Islands, Greece, and Turkey, this species is only known from three females (Baraniak 2003).

### DNA sequence analysis

DNA extracts were prepared from a single leg removed from each of 402 specimens of *Plutella xylostella*. DNA extraction, PCR amplification of the barcode region of COI, and subsequent sequencing followed standard protocols at the Canadian Centre for DNA Barcoding (deWaard et al. 2008). Subsequent analysis focused on the 397 sequence records greater than 500bp in length recovered from these specimens. Sequence divergences were quantified using the Kimura-2-parameter model of nucleotide substitution calculated with the analytical tools on BOLD (www.boldsystems.org). A neighbor-joining (NJ) tree was subsequently constructed with MEGA 5.05 (Tamura et al. 2011).

### Specimen and sequence information

Details on the date and site of collection for each specimen, as well as a photograph are available through the following dataset (dx.doi.org/10.5883/DS-PLUT1). The same DOI provides access to the sequence records, trace files, and primer sequences used for PCR amplification, together with GenBank accession numbers.

### Morphology

Genitalia dissections and slide mounts followed Landry (2007). Pinned specimens were photographed with a Canon EOS 60D camera with a MP-E 65 mm macro lens. They were placed on the tip of a thin plastazote wedge mounted on an insect pin, with the head facing toward the pin and the fringed parts of the wings facing outward. This ensured that there was nothing between the fringes and the background. Lighting was provided by a ring of 144 LEDs covered with a white diffuser dome (Fisher 2012). The camera was attached to a re-purposed stereoscope fine-focusing rail. Sets of 20–35 images in thin focal planes were taken for each specimen and assembled into deep-focused images using Zerene Stacker and edited in Adobe Photoshop.

The configuration of the vinculum-saccus in *Plutella* male genitalia makes it difficult to spread the genitalia open in the standard manner for slide mounting without causing significant distortion. As a result, the differences between *Plutella australiana* and *Plutella xylostella* are not readily apparent if standard mounts are attempted, even if the unrolling technique is employed. To display them properly, the different parts of the male genitalia were separated and temporarily mounted in lactic acid on slides under cover slips raised with vinyl props as wedges to prevent distortion or flattening. After photography, genitalia parts were permanently embedded in Euparal. Genitalia were photographed with a Nikon DS-Fi1 digital camera mounted on a Nikon Eclipse 800 microscope at magnifications of 100×. Nikon’s NIS 2.3 Elements was used to assemble multiple photos of different focal planes into single deep-focus images.

### Specimen depositories

AMSAustralian Museum, Sydney, New South Wales, Australia

ANICAustralian National Insect Collection, CSIRO, Canberra, Australia

ASCUAgricultural Scientific Collections Unit, Orange Agricultural Institute, Orange, New South Wales, Australia

BIOUGBiodiversity Institute of Ontario, University of Guelph, Guelph, Ontario, Canada

BMNHThe Natural History Museum, London, UK

CNCCanadian National Collection of Insects, Arachnids, and Nematodes, Ottawa, Ontario, Canada

USNMNational Museum of Natural History, Smithsonian Institution, Washington, D.C., USA

ZMUCZoological Museum, University of Copenhagen, Copenhagen, Denmark

## Results

### Molecular divergences

**Figure 1.**
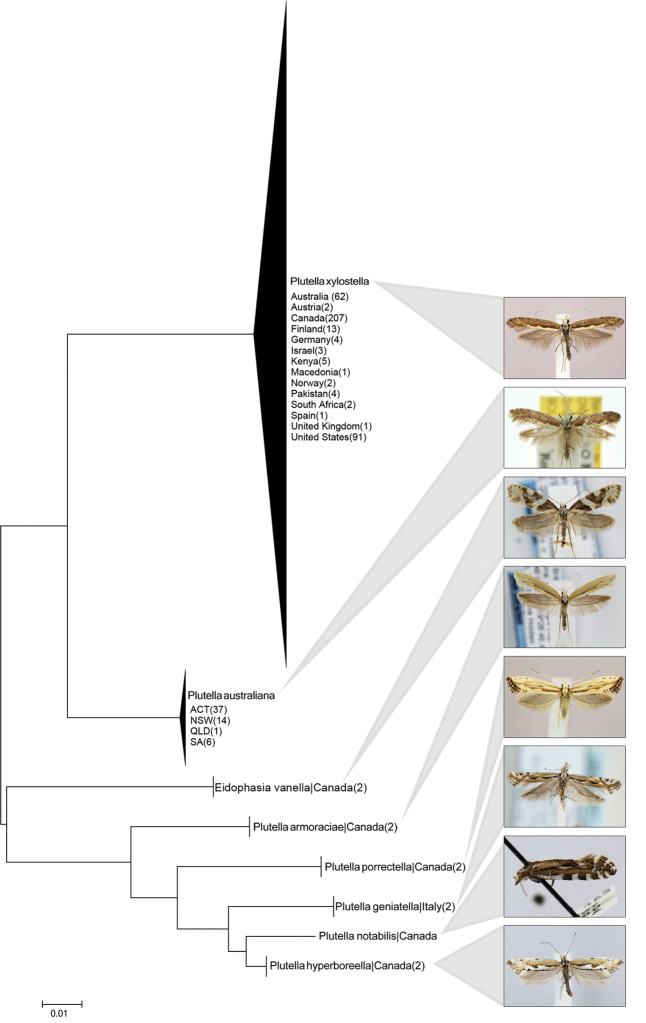
NJ tree based on K2P distances for the barcode region of the cytochrome *c* oxidase I gene among seven species of the genus *Plutella* and one member of the closely allied genus *Eidophasia*. Because of the large number of specimens for both *Plutella xylostella* and *Plutella australiana*, the tree nodes have been collapsed and specimen records are plotted by state for Australia and by country of origin in other cases. The bracketed numerals indicate the number of specimens from each site. The type specimen of *Plutella karsholtella* is reassigned to *Plutella xylostella* and is the only specimen of this species from Spain (Canary Islands).

Analysis revealed that individuals of the five non-Australian species of *Plutella* and the species of *Eidophasia* each formed a distinct sequence cluster in the NJ tree (Figure 1). Interspecific divergences between pairs of these taxa averaged 9.5% and ranged from 2.2%–14.0%. The lowest mean divergence value was between *Plutella notabilis* Busck and *Plutella hyperborella* Strand, while the greatest was between *Plutella xylostella* and *Plutella geniatella* Zeller (Table 1). Because of their deep divergence, each species was assigned to a different Barcode Index Number (BIN) (Ratnasingham and Hebert 2013). No intraspecific sequence variation was detected in the six species included for comparison, but the 397 specimens originally assigned to *Plutella xylostella* were separated into two clusters with 8.6% sequence divergence. Members of one cluster derived from Asia, Australia, Europe, and North America, while those in the other cluster were only collected in Australia. Because subsequent morphological studies indicated clear differences in genitalia between specimens in these two groups (see below), the sequence results were reconsidered presuming that the two clusters represented different species. The broadly distributed lineage was undoubtedly true *Plutella xylostella*, while the other lineage represents an overlooked species that is described below as *Plutella australiana*. Under this model, intraspecific divergence averaged 0.7% in *Plutella xylostella*, and 0.1% in *Plutella australiana*. Sequence analysis also indicated that the holotype female of *Plutella karsholtella* possessed a barcode sequence that was identical to a prevalent haplotype in *Plutella xylostella*.

**Figure 2.**
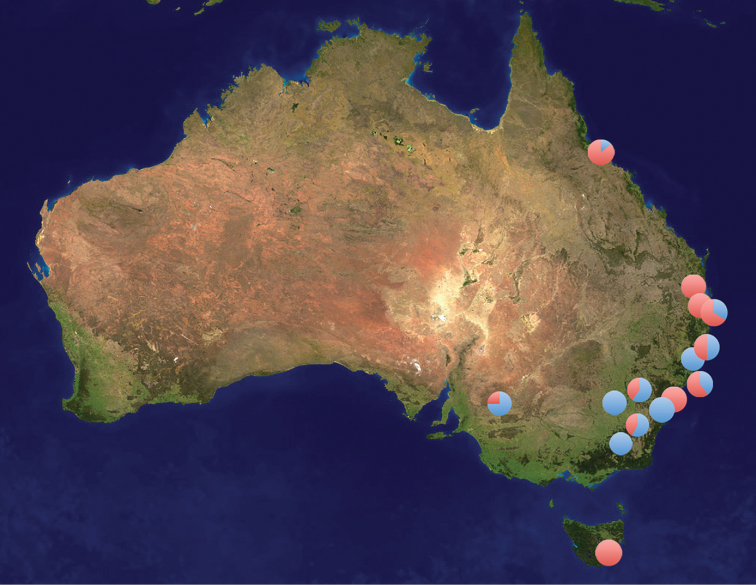
Sites in Australia where specimens of *Plutella xylostella* (red) and *Plutella australiana* (blue) have been collected. The pie diagrams show the proportion of the two species at each site. These records only include specimens identified through DNA barcode analysis.

**Table 1.** Mean sequence divergences (K2P) for the barcode region of the COI gene for seven members of the genus *Plutella* and one member of the closely allied genus *Eidophasia*.

	***Plutella armoraciae***	***Plutella geniatella***	***Plutella hyperboreella***	***Plutella xylostella***	***Plutella notabilis***	***Plutella porrectella***	***Eidophasia vanella***
***Plutella geniatella***	0.071						
***Plutella hyperboreella***	0.050	0.040					
***Plutella xylostella***	0.120	0.140	0.126				
***Plutella notabilis***	0.066	0.043	0.022	0.138			
***Plutella porrectella***	0.079	0.071	0.058	0.138	0.066		
***Eidophasia vanella***	0.098	0.121	0.107	0.115	0.110	0.124	
***Plutella australiana***	0.091	0.098	0.085	0.086	0.088	0.096	0.105

### Species description

#### Plutella australiana

Landry & Hebert sp. n.

http://zoobank.org/20416523-A949-4784-BB18-00A8BA208B6D

http://species-id.net/wiki/Plutella_australiana

Barcode Index Number: BOLD:AAC6876

Figs 3–9 , 17, 19, 21 , 23, 25, 27 , 29, 31

##### Material examined.

Thirty males and 22 females were included in the type series. Five additional specimens were also barcoded but excluded from the type material due to their poor condition.

##### Type material.

Holotype ♂, specimen # BIOUG00844-C06, labelled as follows: [label1] “Subset of: LOT# L#2010AUS-0039 | AUS: New South Wales [sic]: Canberra; Cook | 35.2612°S, 149.0591°E 632m asl 15-Oct-10 | coll. Christy Carr, Paul Hebert, Stephanie Kirk, | Jaclyn McCormick, Jayme Sones”; [label2, pale yellow] “Barcode of Life | DNA voucher specimen | Sample ID BIOUG00844-C06 | BOLD Proc. ID: PHLCA1136-11”; [label3, pale green] “genitalia slide | JFL1731 [male symbol]”; [label4, orange] “HOLOTYPE | Plutella | australiana | J.-F. Landry & Hebert”. Genitalia slide JFL1731. Condition of specimen: double-mounted, wings partly spread, left antenna missing, right hind and left mid- and hind legs removed for DNA barcoding. Deposited in ANIC.

PARATYPES: 29 males, 22 females. **Australian Capital Territory**: Canberra, Cook, 8 Moss Street, 35.261°S, 149.059°E, alt. 632 m, UV light, C. Carr, P.D.N. Hebert, S. Kirk, J. McCormick, J. Sones: 1♂, 1.X.2010, specimen # BIOUG00792-E09 (CNC); 2♂, 6.X.2010, specimen # BIOUG00831-A04 (ANIC), BIOUG00831-H06 (BIOUG); 5♂, 7.X.2010–8.X.2010, specimen # BIOUG00788-B01 (ANIC), BIOUG00788-F08 (ANIC), BIOUG00788-F11 (slide JFL1730) (CNC), BIOUG00788-F12 (ANIC); 1♀, 8.X.2010, specimen # BIOUG00829-H10 (CNC); 1♂, 9.X.2010, specimen # BIOUG00843-C02 (BIOUG); 1♂, 2♀, 15.X.2010, specimen # BIOUG00844-A09 (CNC), BIOUG00844-C03 (AMS), BIOUG00844-G03 (ANIC); 1♂, 2♀, 18.X.2010–20.X.2010, specimen # BIOUG00788-G04, slide JFL1740 (CNC), BIOUG00788-G06, slide JFL1736 (CNC), BIOUG00788-G05 (CNC); 1♂, 25.X.2010, specimen # BIOUG00788-H07 (CNC); 1♀, 27.X.2010, specimen # BIOUG00790-G12 (ANIC). Same locality, collected by P.D.N. Hebert: 2♂, 22.III.2011, specimen # BIOUG01025-G05 (ZMUC), BIOUG01025-G06 (CNC); 1♂, 1♀, 10.XI.2011, CCDB-12828-G04 (ZMUC), CCDB-12828-F10 (AMS); 1#m 12.XI.2011, specimen # BIOUG02125-G06 (CNC); 3♂, 1♀, 13.XI.2011, specimen # BIOUG02127-F12 (ANIC), BIOUG02127-G10 (BMNH), BIOUG02127-H01 (ANIC), BIOUG02127-H03 (ANIC); 1♂, 1♀, 16.XI.2011, CCDB-15380-G10 (BIOUG), CCDB-15380-E08 (AMS); 1♀, 18.XI.2011, BIOUG02123-E08 (USNM); 1♀, 23.X.2011, specimen # BIOUG02109-B09 (BMNH); 1♀, 24.X.2011, specimen # BIOUG02109-C07, slide JFL1741 (ANIC); 1♀, 5.XI.2011, specimen # BIOUG02112-F11 (AMS); 1♀, 6.XI.2011, specimen # BIOUG02108-C09, slide JFL1737 (CNC). Same locality, collected by P.D.N. Hebert, R. Labbee, V. Levesque-Beaudin, J. McCormick, J. Sones, J. Webb: 1♀, 29.III.2011–12.IV.2011, specimen # BIOUG01172-G03, slide JFL1738 (ANIC). Canberra, CSIRO property, 35.275°S, 149.111°E, alt. 588 m: 1♀, 14.XI.2011–21.XI.2011, Malaise trap, P.D.N. Hebert, specimen # BIOUG02239-A02 (BIOUG). **New South Wales**: Byron Bay, 28.658°S, 153.622°E, alt. 13 m: 1♀, 30.XII.2007, P.D.N. Hebert, specimen # 07-NSWBB-0046, slide JFL1684 (CNC). 2800 Pinnacle Rd., Lot 58, 33.297°S, 149.075°E, alt. 920 m., 1♀, 3.III.2005, H. Loecker, specimen # 05-NSW-00731 (ASCU). Orange, 353 Pinnacle Rd., UV light trap, 33.297°S, 149.075°E, 2♂, 1♀, 26.X.2010, H. Loecker, specimen # ww04709–ww04711 (ASCU). Smiths Lake, 32.377°S, 152.504°E, 1♂, 1♀, 24.XII.2010–24.XII.2010, P.D.N. Hebert, specimen # BIOUG00987-B02 (ANIC), BIOUG00987-E12 (slide JFL1735) (CNC). Weddin Mt. National Park/Bimbi State Forest, Grenfell, nr. “Seatons Farm”, 33.913°S, 147.947°E, at light, 1♀, 9.XI.2007, H. Loecker, specimen # AM 2272, slide JFL1739 (ASCU). Hat Head, 31.063°S, 153.052°E, alt. 36.58 m., 2♂, 28.XII.2008, P.D.N. Hebert, specimen # 08-NSWHH-1277 (slide JFL1689) (ANIC), 08-NSWHH-1340 (slide JFL1690) (CNC). **South Australia**: 1 km N Border Cliffs, near the banks, Renmark, 34.024°S, 140.89°E, 4♂, 25.XI.2011, P.D.N. Hebert, UV light trap, specimen # BIOUG02248-G03 (slide JFL1732) (CNC), BIOUG02248-F12 (ANIC), BIOUG02248-G01 (ANIC), BIOUG02248-G04 (USNM). Lyrup Forest Reserve, 34.274°S, 140.64°E, 1♀, 8.XII.2011, P.D.N. Hebert, UV trap by lake, specimen # BIOUG02246-B09 (ANIC). Pike Creek Woolshed, 34.278°S, 140.711°E, 1♂, 6.XII.2011, P.D.N. Hebert, mercury vapor light, specimen # BIOUG02120-H01 (ANIC).

##### Additional specimens barcoded, but not included in the type series.

**Australian Capital Territory**: Canberra, Manuka, 35.278°S, 149.166°E, 1 ex. (abdomen missing), 16.XII.2005, P. Hebert, specimen # 05-ACTC-285 (BIOUG). **New South Wales**: 2800 Pinnacle Rd., Lot 58, 33.297°S, 149.075°E, 1♀, 1 ex. (abdomen missing), 24.II.2005, P.D.N. Hebert, specimen # 05-NSW-00732 (ASCU). Ellenborough, Tom’s Creek Retreat, 31.459°S, 152.476°E, 1 ex (abdomen missing), 17.XII.2005, P.D.N. Hebert, specimen # 06-NSWE-00800 (BIOUG). Kosciuszko National Park, Charlottes Pass, 36.26°S, 148.2°E, alt. 1844 m., 1♂, 1♀, 08–09.III.2009, E.D. Edwards, specimens # am10299, am10372 (ANIC). **Queensland**: Townsville, Hermit Park, 19.283°S, 146.801°E, 1 ex., 01.X.2010, G. Cocks, specimen # gvc15526-1L (AMS).

##### Diagnosis.

**Figures 3–16.**
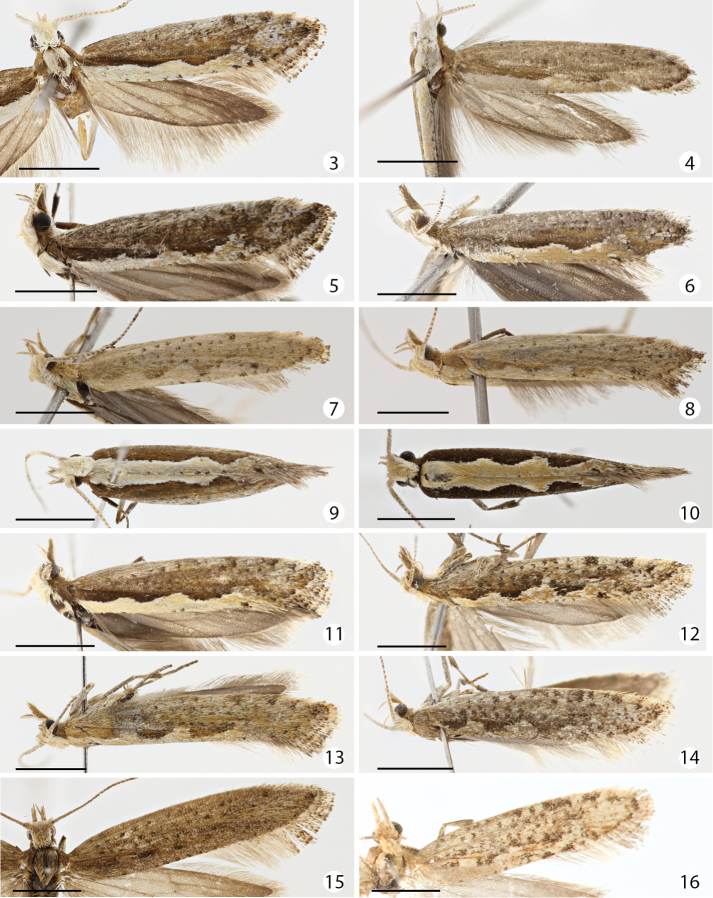
Dorsal aspect of forewings of *Plutella australiana* and *Plutella xylostella*. SpecimenID (sample ID) in parentheses. Scale bar = 2 mm. **3** *Plutella australiana*, male holotype, Australia: Canberra (BIOUG00844-C06) **4** *Plutella australiana*, female paratype, Australia: New South Wales, Bimbi State Forest (AM 2272) **5** *Plutella australiana*, male paratype, Australia: Canberra (BIOUG00788-F11) **6** *Plutella australiana*, female, Australia: Canberra (BIOUG02123-E08) **7** *Plutella australiana*, female, Australia: Canberra (BIOUG02112-F11) **8** *Plutella australiana*, female, Australia: Canberra (BIOUG02108-C09) **9** *Plutella australiana*, male, Australia: Canberra (CCDB-15830-E08) **10** *Plutella xylostella*, male, Australia: Canberra (CCDB-12828-E05) **11** *Plutella xylostella*, male, Australia: Canberra (BIOUG02113-F05) **12** *Plutella xylostella*, female, Australia: Canberra (BIOUG01172-A09) **13** *Plutella xylostella*, female, Australia: Canberra (CCDB-12828-G07) **14** *Plutella xylostella*, female, Australia: Canberra (CCDB-12828-H01) **15** *Plutella xylostella*, female, Canada: Québec (CNCLEP00098486) **16** *Plutella xylostella*, female holotype of *Plutella karsholtella*, Canary Islands: Tenerife (ZMUC00401145).

In external appearance *Plutella australiana* is indistinguishable from *Plutella xylostella*. Both species exhibit significant, overlapping variation in forewing pattern (Figs 3–16). Most specimens of both species have the pale, scalloped band along the hind/dorsal margin typically used to recognize *Plutella xylostella*. That band varies from strongly marked to nearly indistinct (the latter particularly so in females) in both species. Here we illustrate only a selection of the variants, but intermediates in amount of dark specking and spotting, fading of scalloped dorsal band, and intensity of brown colouration, exist among specimens of both examined. No reliable external difference was observed that permits the separation of the two species. Genitalia must be examined and they afford several good characters.

**Figures 17–22.**
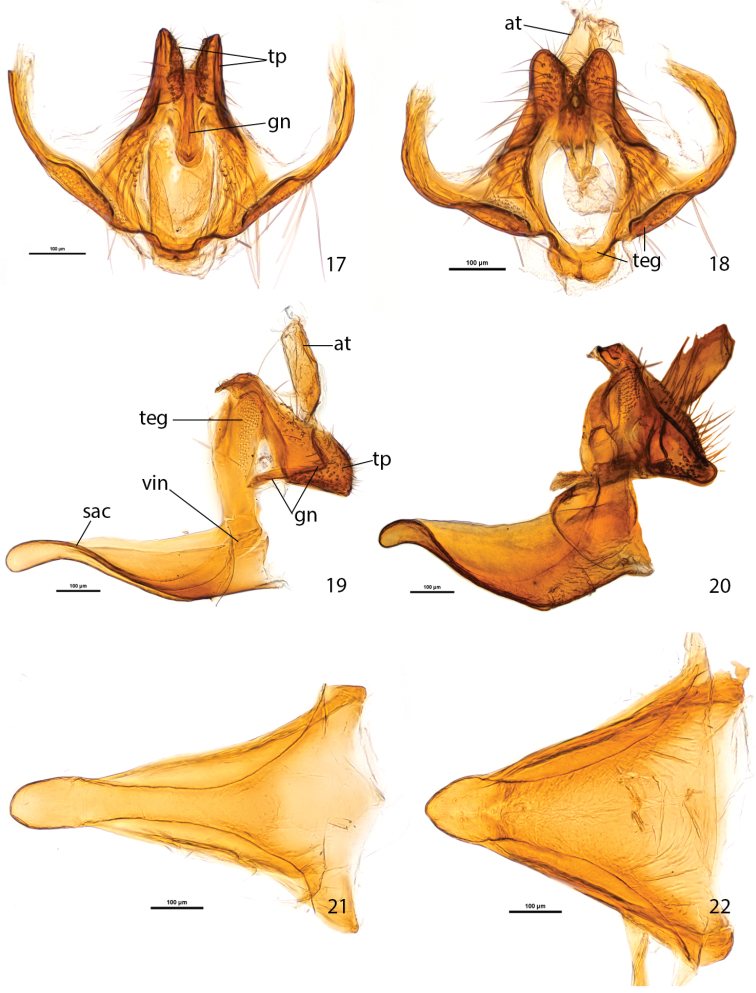
Male genitalia of *Plutella australiana* and *Plutella xylostella*. **17–18** tegumen-gnathos, ventral aspect **17** *Plutella australiana* (slide JFL1730, specimen BIOUG00788-F11) **18** *Plutella xylostella* (slide JFL1729, specimen BIOUG02113-A06). 19–20, tegumen-uncus-gnathos-vinculum complex, lateral aspect, valvae and phallus removed **19** *Plutella australiana* (slide JFL1732, specimen BIOUG02248-G03) **20** *Plutella xylostella* (slide JFL1733, specimen BIOUG02248-G02). **21–22** vinculum–saccus, ventral aspect **21** *Plutella australiana* (slide JFL1730) **22** *Plutella xylostella* (slide JFL1729). Scale bar = 100µ; all to same scale. at = anal tube; gn = gnathos; sac = saccus; teg = tegumen; tp = teguminal process; vin = vinculum.

**Figures 23–28.**
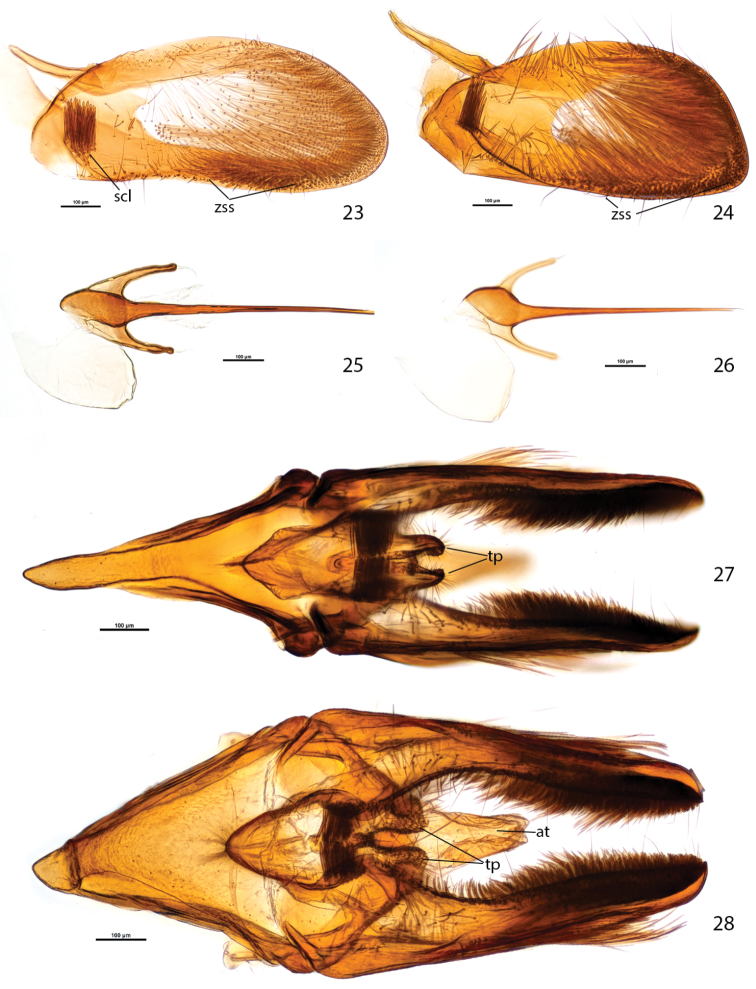
Male genitalia of *Plutella australiana* and *Plutella xylostella*. 23–24, valva, inner aspect **23** *Plutella australiana* (slide JFL1689, specimen 08-NSWHH-1277) **24** *Plutella xylostella* (slide JFL1733, specimen BIOUG02248-G02). 25–26, phallus, dorsal aspect **25** *Plutella australiana* (slide JFL1690, specimen 08-NSWHH-1340) **26** *Plutella xylostella* (slide JFL1688, specimen 06-TASB-01769). 27–28, genitalia with phallus removed, ventral aspect **27** *Plutella australiana* (slide JFL1735, specimen BIOUG00987-E12) **28** *Plutella xylostella* (slide JFL1734, specimen 09-NSWHH-1674). Scale bar = 100µ; all to same scale. at = anal tube; scl = sacculus; tp = teguminal process; zss = zone of spiniform setae.

In *Plutella australiana*, the male genitalia appear overall more slender than in *Plutella xylostella*, particularly if viewed ventrally (Figs 27–28). The most easily observed difference involves the shape of the vinculum-saccus (Figs 19–22): in *Plutella australiana* it is slender with a slight medial constriction, a more protruded and inflated anterior apex, and is about 1.5× as long as wide; in *Plutella xylostella* it has a broader, more chunky aspect and in profile, is deeply concave, and is about as long as wide. The teguminal processes (Figs 17–18) are more slender and slightly separated medially in *Plutella australiana*, whereas they are broader and medially contiguous in *Plutella xylostella*. The valva (Figs 23–24) is evenly rounded with a slight sinuation in the ventral margin, and a zone of spiniform setae that is restricted to the medial area in *Plutella australiana*; whereas its ventro-distal margin is more or less distinctly angled and the zone of spiniform setae extends all the way to the angled apex in *Plutella xylostella*.

**Figures 29–32.**
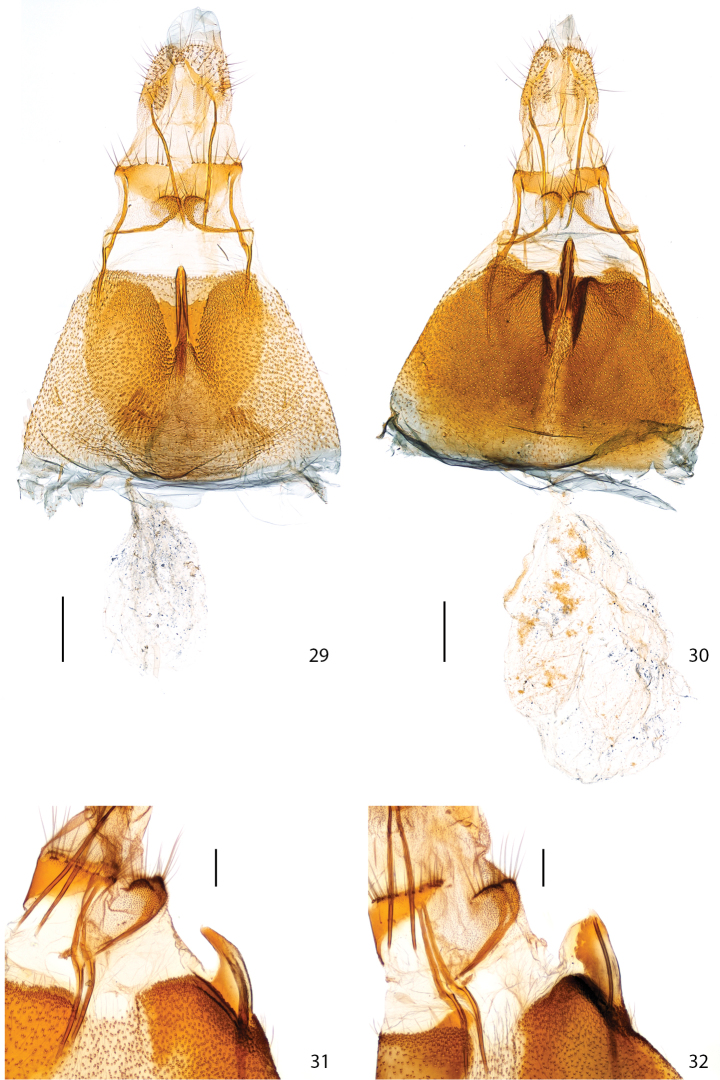
Female genitalia. **29** *Plutella australiana*, ventral aspect (slide JFL1684, specimen 07-NSWBB-0046) **30** *Plutella xylostella*, ditto (slide JFL1685, specimen 07-NSWBB-0144) **31** *Plutella australiana*, lateral aspect of antrum projection (slide JFL1736, specimen BIOUG00788-G06) **32** *Plutella xylostella*, ditto (slide MIC6811, specimen BIOUG02113-D10). Scale: Figs **29–30** = 200µ; Figs **31–32** = 100µ.

Female genitalia: In *Plutella australiana*, abdominal sternum 7 (S7) has a heart-shaped melanized area surrounding the antrum and it has a flat surface; the apex of the tubular projection of the antrum is barely extended beyond the posterior margin of S7 (0.15× length of S7) when viewed ventrally (Fig. 29), and has a constricted, curved apical half when viewed laterally (Fig. 31). In *Plutella xylostella*, the area of S7 surrounding the antrum is bordered by markedly raised pair of folds of the S7 wall which form two conical projections bracing the tubular projection of the antrum; the apex of the tubular projection of antrum is extended further out beyond posterior margin of S7 (0.5× length of S7) when viewed ventrally (Fig. 30), and is evenly broad and straight when viewed laterally (Fig. 32). The corpus bursae is proportionally smaller and about equal in length to S7 in *Plutella australiana*, whereas in *Plutella xylostella* it is proportionally larger and about 1.5× the length of S7.

##### Description.

**Male** (Figs 3, 5, 9). Head off-white, vertex pale greyish brown, area behind eye and beneath ocellus greyish brown. Labial palpus porrect, segment 2 with forward-directed triangular, pale greyish brown tuft, leading edge white; segment 3 upturned, as long as 2, greyish white to greyish brown. Antenna about two-thirds length of forewing; scape with dense off-white to pale greyish brown pecten; flagellum dorsally off-white with a few scattered brown rings in distal half. Mesoscutum off-white. Tegulae greyish brown to brown. Forewing upper surface with costal region brownish grey with sparse, darker speckles; medial and fold region brown to buff-brown; dorsal region off-white to pale buff, sinuous margin with two or three scallops and lined with thin dark brown line, forming a dorsal band of two or three diamonds when wings are folded, in several specimens with dorsal margin lined with a few scattered dark brown dots; apical area paler and mottled with a mix of pale grey, brown, and black suffusion; apical fringe with alternating white and dark brown thin bands.

**Female** (Figs 4, 6, 7, 8). As male except colouration more variable, with several individuals paler overall and with subdued contrast from brownish buff to pale whitish grey, tegula off-white, scalloped dorsal region of forewing indistinct, costal and disc area with dark speckles.

Forewing length: males, 5.4mm–6.9mm (mean 6.1mm, n=30); females, 5.6mm–6.9mm (mean 6.1mm, n=22).

**Male genitalia** (Figs 17, 19, 21, 23, 25, 27) (6 preparations examined). Abdominal tergite 8 (T8) subquadrate, anteriorly with round emargination and protruded antero-lateral corners. Pleural lobes large, about as long as T7+T8, their inner margin edged with thin, ribbon-like sclerotization. Coremata present, 3.5× length of T8. Tegumen a narrow, transverse arch with very narrow dorsal rim, anterior margin laterally notched, pedunculi fused with dorsal arms of vinculum. Uncus absent. Teguminal processes developed as pair of heavily sclerotized, setose lobes projected to one-third of valva length; in profile with dorsal edge broadly rounded and ventral edge straight; in ventral view outline conical with slight outward curvature. Anal tube with lightly sclerotized distal portion about length of teguminal processes. Gnathos a narrow band extended and bent downward at right angle between teguminal processes with apex pointed anteriorly. Vinculum arms dorsally extended and fused with tegumen, ventrally extended into slender, triangular, transversely arched and ventrally concave saccus; in ventral view saccus about 1.5× as long as wide, middle portion slightly constricted, in profile about 5× as long as high. Valva subelliptical in outline, costa and apex evenly rounded, ventral margin slightly concave, length/width about 2.3; spiniform setae of ventral margin arranged in stretched cluster restricted to medial area; sacculus situated in antero-ventral area, with tight cluster of spiniform setae, which medially abut each other below socii when valvae are closed; outer wall of valva with transparent suboval “window” bearing two tufts of long, fine scales, one in antero-ventral third, the other in dorso-distal third. Phallus thin, sharply pointed, needle-like, with bulbous base braced by pair of posteriorly directed, hook-like processes; bulbus ejaculatorius crescentic in outline, about the size of bulbous base.

**Female genitalia** (Figs 29, 31) (7 preparations examined). Sternite 7 (S7) with medio-posterior, heart-shaped, sclerotized area that is distinctly delineated from less melanized antero-lateral areas, posterior emargination forming channel surrounding tubular medial projection of antrum bearing apical ostium bursae, sides of channel flat, only medial anteriormost portion of channel slightly raised around base of antrum; tubular projection 0.4× length of S7, in ventral aspect with apex only slightly extended beyond posterior margin of S7 (0.15× length of S7), in lateral view with distal portion constricted and upcurved. Ductus bursae thin, delicate, anterior two-thirds membranous, posterior third (section inside antrum) sclerotized. Corpus bursae slightly shorter (0.9) than S7, thinly membranous, delicate, without signa. Posterior apophyses subequal in length to anterior ones. Anterior apophyses with a thin ventral branch extended transversally across S8 to lamella postvaginalis in middle; lamella postvaginalis composed of paired crescentic pads covered with sensilla trichodea and distally setose. Tergite 8 with transversely sclerotized distal third on which base of posterior apophyses are inserted, posterior margin lined with setae. Sternite 8+ovipositor subequal in length to S7.

**Note about male genitalia.** The gnathos of *Plutella xylostella* has not been described in recent publications (Robinson and Sattler 2001, Baraniak 2003). It is present in both *Plutella xylostella* and *Plutella australiana* but difficult to see, especially in standard preparations in which the genitalia is mounted whole and flattened, because it is a narrow band wedged between the two teguminal processes, and it projects inwards (see Figs 17–20). Clarke (1971: 173) stated vaguely “gnathos rather involved, with sclerotized plates on each side”, which actually described the teguminal processes more than the gnathos. He went so far as to say that the species was so well known that the description of its genitalia appeared superfluous! The male coremata are tightly associated with the anterior edge of the tegumen-vinculum arch and difficult to keep attached to the pleural lobes during the dissection process.

The term ‘teguminal processes’ has been used by Kyrki (1984) to designate the paired, setose structures that extend from the posterior margin of the tegumen. These have been termed ‘socii’ by some authors (e.g. Common 1990). Dugdale et al. (1998) stated that these processes were gnathal rather than teguminal, but did not provide supporting evidence. Kuznetsov and Stekolnikov (1976) called them socii in *Plutella xylostella* but, contrastingly, termed ‘gnathos’ topographically and functionally similar paired structures in *Eidophasia messingiella* (Fischer von Röslerstamm), a genus often related to *Plutella*. Our own examination revealed only a faint suggestion of suture between those lobes and the transversely narrow tegumen, as well as between the tegumen and the lateral arms of the gnathos. However, all these structures appear to be fused together. What seems clear to us is that the morphology of this region of the male genitalia has not been documented in sufficient detail to interpret these structures unequivocally. Therefore we prefer to use the more morphologically neutral term ‘teguminal processes’.

##### Derivation of specific epithet.

This species name reflects the current restriction of this taxon to Australia. It is a noun in apposition.

##### Distribution.

*Plutella australiana* is so far known only from eastern Australia, in contrast to *Plutella xylostella* which is cosmopolitan in distribution. *Plutella australiana* and *Plutella xylostella* appear to have largely overlapping distributions within Australia as both species were collected in the ACT, NSW, QLD, and SA (Figure 2). They were collected together and on the same dates around Canberra on several occasions (Mar 2011, Apr 2011, Oct 2010 and 2011, Nov 2011), indicating that their adult flight periods and habitat requirements overlap. *Plutella australiana* may occur in other parts of Australia with the lack of records reflecting gaps in collecting. Although current records suggest that *Plutella australiana* is absent from Tasmania, further sampling is also required to confirm this fact. The two species appear to be roughly similar in abundance based on current records with 62 *Plutella xylostella* and 57 *Plutella australiana* among the haphazardly collected Australian specimens that have been sequenced.

##### Type locality.

Australia, Australian Capital Territory, Cook, 35.2612°S, 149.0591°E.

##### Host plant.

*Plutella xylostella* is thought to feed on a wide variety of cruciferous plants in Australia, including native and introduced species. However, Australian records are in question because of the past oversight of *Plutella australiana*. As a result, the host plants of both species are uncertain.

#### Plutella xylostella

(Linnaeus, 1758)

http://species-id.net/wiki/Plutella_xylostella

Barcode Index Number: BOLD:AAA1513

Figs 10–16 , 18, 20, 22 , 24, 26, 28 , 30, 32

Plutella karsholtella Baraniak, 2003: 31. New synonymy. Type locality: Canary Islands, Tenerife. Holotype in ZMUC. Barcoded.

##### Remarks.

Baraniak (2003) described *Plutella karsholtella* from three female specimens based on minor differences in genitalia from *Plutella xylostella*. The main difference (given in his diagnosis) is that the distal portion of the ductus bursae has a curve at the level of the antrum when viewed laterally. There are two drawings of the female genitalia in Baraniak (2003), one showing the ventral aspect, the other in lateral aspect, but it is not indicated what preparations or specimens they are based on, nor whether both were drawn from the same specimen. Considering that the two paratypes are from localities widely distant from the type locality (one is from northwestern Turkey, the other from Greece) and that the difference from *Plutella xylostella* is slight, it would have been important to indicate the stability of this trait. The similarity of the holotype barcode with a common haplotype of *Plutella xylostella* and the single minor difference in female genitalia (male genitalia unknown) suggest that it is synonymous with the latter and we consider it so here. We omit the suite of other previously well-established junior synonyms of *Plutella xylostella*, which can be found in Robinson and Sattler (2001).

The colouration of *Plutella xylostella* has been characterized as variable, with paler individuals in xeric regions (Robinson and Sattler 2001). Our examination of many specimens from Asia, Australia, Europe, and North America showed that much of the forewing variation appears restricted to females. Males are relatively constant in having the typical forewing pattern with a strongly defined, ochre or cream-coloured, scalloped dorsal fascia contrasting markedly with the brown anterior two-thirds. Females display significant individual variation deviating from this pattern, from a dorsal fascia that is more subdued to one that is indistinct or nearly lacking (Figs 12–16).

In a taxonomic review of Hawaiian *Plutella*, Robinson and Sattler (2001) described two morphologically indistinguishable ‘host races’ of *Plutella xylostella*, reared from larvae consuming the fruits (rarely the leaves) of caperbush (*Capparis*, Capparaceae). The recognition of two separate races with the same, albeit unusual, host was geographical, each being restricted to an island: ‘host-race 1’ found on Oahu was characterized as having a forewing pattern typical of “faded or at best weakly indicated” *Plutella xylostella*; whereas ‘host-race 2’ found on the big island of Hawaii was described as “unusual very pale (…) white to cream with faded yellow markings”. They did not find significant genitalia differences from typical *Plutella xylostella*, which also occurs in the Hawaiian archipelago where it has been reared from several Brassicaceae. The colour differences that they describe for the host races appear to fall within the known variation of *Plutella xylostella* elsewhere and may not be diagnostically significant.

At least one of these Hawaiian races was included in a previous study of mtDNA variation in *Plutella xylostella* (as undescribed *Plutella* ‘UPA’ by Chang et al. (1997)). However, the sequenced specimens were without host plant record (not reared) and no vouchers were retained so their identity cannot be verified. Their short sequences (GenBank accession numbers AF019041 for *Plutella* ‘UPA’ and AF019042 for *Plutella* ‘UPB’) overlap the 3’ half of the barcode region and, when compared to our results, are more than 10% divergent from the *australiana*–*xylostella* cluster, suggesting no conspecificity with either.

## Discussion

*Plutella xylostella* has long been regarded as a very common and widely distributed species within Australia (Nielsen et al. 1996). The present reevaluation of its taxonomic status was motivated by the results of DNA barcode analysis which revealed that its Australian populations included two lineages showing 8.6% sequence divergence. Because prior studies have indicated that levels of intra-specific variation rarely exceed 2% in Lepidoptera (Hajibabaei et al. 2006, Hebert et al. 2009, Hausmann et al. 2011), this discovery strongly suggested that two species were present. Subsequent morphological examination confirmed the presence of clear differences in genitalia between specimens of the two taxa, motivating recognition of the Australian lineage as a new species. Although this study has led to the discovery of one cryptic species, it has also provided evidence that another species in the genus, *Plutella karsholtella*, is a junior synonym for *Plutella xylostella*. We base this conclusion on both its barcode identity with one of the commonest haplotypes in *Plutella xylostella* and its lack of clear diagnostic morphological features.

The past oversight of the presence of two *Plutella* species in Australia likely explains the regional allozyme variation previously detected in Australian populations of *Plutella xylostella* (Pichon et al. 2006). For example, the congruence between populations near Sydney and those in other nations could be explained if *Plutella xylostella* dominated collections from this locality, while those at the other sites were dominated by *Plutella australiana*. The presence of these two species also has implications for past evaluations of biological control strategies, particularly since both species appear to be abundant and widely distributed in eastern Australia.

There is a need to discover the host plant(s) of *Plutella australiana* to ascertain if it is also a crop pest. If so, its presence represents a new risk to international trade which should be evaluated. Examining known hosts of *Plutella xylostella* and other related *Plutella* may provide useful clues. Although *Plutella xylostella* has been recorded from a wide range of hosts across the world, records from plant families other than Brassicales are uncorroborated, with most being implausible (Robinson and Sattler 2001). However, there are notable exceptions. For example, two ‘host races’ of *Plutella xylostella* in Hawaii (Robinson and Sattler (2001)) have been reared from the fruits of caperbush (*Capparis*, Capparaceae, order Brassicales). Löhr and Rossbach (2001) reported a population of *Plutella xylostella* from Kenya feeding on sugar snap pea, *Pisum sativum* (Fabaceae). Laboratory tests showed that it was bi-directionally cross-fertile with “normal” crucifer-feeding *Plutella xylostella* producing viable offspring. Pea-feeding *Plutella xylostella* also survived on kale (a crucifer). Their study mentioned that the identity of the species was “not in question”, but they did not provide morphological or genetic evidence to support this assertion. It is also noteworthy that *Plutella armoraciae*, a species from northwestern North America that superficially looks like a very pale *xylostella* (but is 12% barcode divergent), feeds on horseradish (*Armoracia*, a perennial Brassicaceae) (Robinson et al. 2000), and *Eidophasia dammersi* (Busck), originally described in *Plutella*, from California, feeds on the perennial *Cleome isomeris* (Cleomaceae), another plant family in the Brassicales. These observations suggest the possibility that the host plant of *Plutella australiana* is not necessarily a Brassicaceae and that the Brassicales should be searched widely to ascertain the hosts for *Plutella australiana*. Capparaceae and Cleomaceae are closely related to Brassicaceae with similar phytochemistry (Stevens 2001, Reveal 2011). The single documented occurrence of *Plutella xylostella* on a non-Brassicale host might considerably broaden the host possibilities, but the uniqueness of this record and its restricted geographical location in Africa require further study. Restricting the search for *Plutella australiana* larvae to Brassicales in Australia might be a more fruitful approach to discover its host.

The high genetic distance among taxa analyzed, and the placement of *Eidophasia vanella* between the *Plutella australiana* – *Plutella xylostella* cluster and other members of *Plutella* suggests that current generic limits need further assessment. Baraniak (2003), studying only the Palearctic fauna and morphology, separated most species formerly in *Plutella* into two separate genera, *Pseudoplutella* Baraniak (monotypic with only *Plutella porrectella* (Linnaeus), and *Plutelloptera* Baraniak (including *Plutella geniatella* and *Plutella hyperboreella* of the present analysis), leaving *Plutella* to comprise only *Plutella xylostella* – *Plutella karsholtella*. Despite his arrangement being based on a cladistic analysis, his genera have not been widely adopted or are treated as subgenera of *Plutella* (Fauna Europaea 2013). Further work should combine morphological and genetic data with global taxon coverage to gain a better understanding of generic boundaries.

## Supplementary Material

XML Treatment for Plutella australiana

XML Treatment for Plutella xylostella
